# HIV, Stigma, and Rates of Infection: Absence of Evidence

**DOI:** 10.1371/journal.pmed.0040054

**Published:** 2007-01-30

**Authors:** Mark Seielstad

Absence of evidence is not evidence of absence. Reidpath and Chan [1] present absolutely no reasons or data that would undermine the hypothesis that stigmatization can increase the risk of HIV spread in populations (nor any in favor of their own contrary hypothesis that stigmatization could halt the spread of HIV). More data to address the question may be needed to establish either hypothesis, but motivations to collect such data should not come from illogical and inflammatory counterhypotheses. In the guidelines for correspondence to *PLoS Medicine*, we are advised that “...letters inciting racial hatred, sexism, or homophobia [will not be published]” [2], but the present authors skirt the commission of such acts very closely. After all, HIV prevalences also differ among races, sexes, and sexual orientations, so the stigmatization of groups with high rates of infection by those with lower rates of infection could be viewed as helpful to the goal of HIV containment. This seems unlikely to be helpful, and would impose a much greater societal cost. This essay is shameful in its intellectual dishonesty and lack of evidence to support its own unappealing counterhypothesis.
